# Modified preputial elevation and cranial translation of the prepuce by transection of the skin caudal to the bulbus glandis for managing chronic idiopathic paraphimosis in dogs: six cases (2021‐2024)

**DOI:** 10.1111/jsap.70038

**Published:** 2025-10-28

**Authors:** I. P. Thyriakis, C. Ververidis, L. Pavlidis, V. Angelou, K. Chatzimisios, L. G. Papazoglou

**Affiliations:** ^1^ Companion Animal Clinic, School of Veterinary Medicine Aristotle University of Thessaloniki Thessaloniki Greece; ^2^ Clinic of Plastic Surgery, School of Medicine Aristotle University of Thessaloniki, Papageorgiou Hospital Thessaloniki Greece

## Abstract

**Objectives:**

To report the signalment, clinical signs, duration of clinical signs, length of penile protrusion, surgical technique (modified preputial elevation and cranial translation of the prepuce), length of preputial advancement, postoperative complications, outcomes and long‐term postoperative follow‐up.

**Materials and Methods:**

Retrospective case series. The medical records of dogs with idiopathic paraphimosis undergoing elevation and cranial translation of the prepuce between 2021 and 2024 were reviewed.

**Results:**

Six dogs with idiopathic paraphimosis were identified. The median duration of paraphimosis was 9.5 months. The median length of the penile protrusion was 2 cm (range: 1 to 3 cm). All dogs recovered uneventfully from surgery. The median translation of the prepuce at surgery was 4 cm (range: 2 to 6 cm). Four minor postoperative complications were observed in three dogs, including serosanguineous discharge from the incision, preputial oedema formation, penile exposure and a small dehiscence. All short‐term complications resolved without treatment. Median follow‐up time was 24 months. All dogs had an excellent outcome. In one dog, 14 months after surgery, a 0.5 cm long intermittent penile protrusion was noted with no clinical significance.

**Clinical Significance:**

Modified preputial elevation and cranial translation of the prepuce could be considered an option for the treatment of idiopathic paraphimosis in dogs.

## INTRODUCTION

The inability to fully retract the glans penis into the preputial cavity is known as paraphimosis, which can be acute or chronic (MacPhail, 2019; Papazoglou & Kazakos, 2002; Yiapanis et al., 2021). Causes include trauma, mating, infection, neoplasms, priapism and developmental defects (Papazoglou & Kazakos, 2002). Chronic paraphimosis may occur when no apparent defects in the preputial orifice are present, possibly due to ineffective preputial muscles, a hypoplastic prepuce or idiopathic causes (Chaffee & Knecht, 1975; Hobson, 2014; Papazoglou, 2001; Papazoglou & Kazakos, 2002). In chronic paraphimosis, dryness, cornification and irritation of the protruded penis, along with excessive licking, traumatization and discomfort, may be encountered, and the condition may be cosmetically unacceptable (Hobson, 2014; MacPhail, 2019). Idiopathic paraphimosis has rarely been reported in dogs (Ndiritu, 1979; Papazoglou, 2001; Somerville & Anderson, 2001).

Acute paraphimosis requires conservative management with lubrication, hot/cold therapy using hyperosmolar solutions, to reduce penile swelling and facilitate manual reduction of the penis within the preputial cavity. Surgery should be considered if retraction is impossible (Papazoglou & Kazakos, 2002). Cranial preputial advancement and/or phallopexy have been reported to correct chronic paraphimosis in dogs (Papazoglou, 2001; Somerville & Anderson, 2001). Cranial preputial advancement entails removing a crescent‐shaped piece of skin cranial to the prepuce, excision of the preputial muscles and reapposition of the muscles with sutures by incorporation of the rectus abdominis muscle in the closure (Papazoglou, 2001). Preputial advancement was successful when the length of the exposed penis was less than 1.5 cm (Papazoglou, 2001). Phallopexy can also be by creating a permanent adhesion between the penis dorsally and the corresponding preputial mucosa to successfully address chronic paraphimosis in three dogs (Somerville & Anderson, 2001). A combination of these two techniques has also been described in the treatment of a dog with chronic paraphimosis (Wasik & Wallace, 2014). Partial penile amputation has also been used for the treatment of chronic inflammation and irritation secondary to chronic paraphimosis (Ritson et al., 2023). Recently, preputial elevation and cranial translation of the prepuce by transection of the skin caudal to the bulbus glandis (modification 1) and/or by releasing the penis from the laminal interna (modification 2) were reported in cadaveric dogs for addressing long penile protrusions (Yiapanis et al., 2021). Recently, a Y‐V‐shaped preputial advancement plasty with cranial translation of the penile sheath was successfully used for the management of 2 dogs with paraphimosis (Schreiber et al., 2023). At the time of writing, no published studies have described preputial elevation and cranial translation of the prepuce in the treatment of clinical cases with chronic paraphimosis.

The aim of this study was to report the surgical technique as described by Yiapanis et al. (2021), complications and long‐term follow‐up of six dogs that underwent a modified preputial elevation and cranial translation of the prepuce by transection of the skin caudal to the bulbus glandis (MPECT) for managing chronic idiopathic paraphimosis.

## MATERIALS AND METHODS

### Study design and data extraction

The study was approved by the graduate programme coordinating committee of the School of Veterinary Medicine. Client consent was obtained at the time of surgery and retrospectively for follow‐up information. Search terms included dogs, paraphimosis, idiopathic paraphimosis and preputioplasty. The medical records of six dogs with idiopathic paraphimosis that were referred to a single referral institution for further treatment (Clinic of Companion Animals) and underwent preputial elevation and cranial translation of the prepuce were reviewed between 2021 and 2024. The data collected included signalment, clinical examination findings, duration of clinical signs before presentation, length of the prolapsed penis during clinical examination in a standing relaxed position, surgical technique (MPECT), length of preputial translation, postoperative complications, outcomes and long‐term postoperative follow‐up.

### Surgical technique

**FIG 1:**
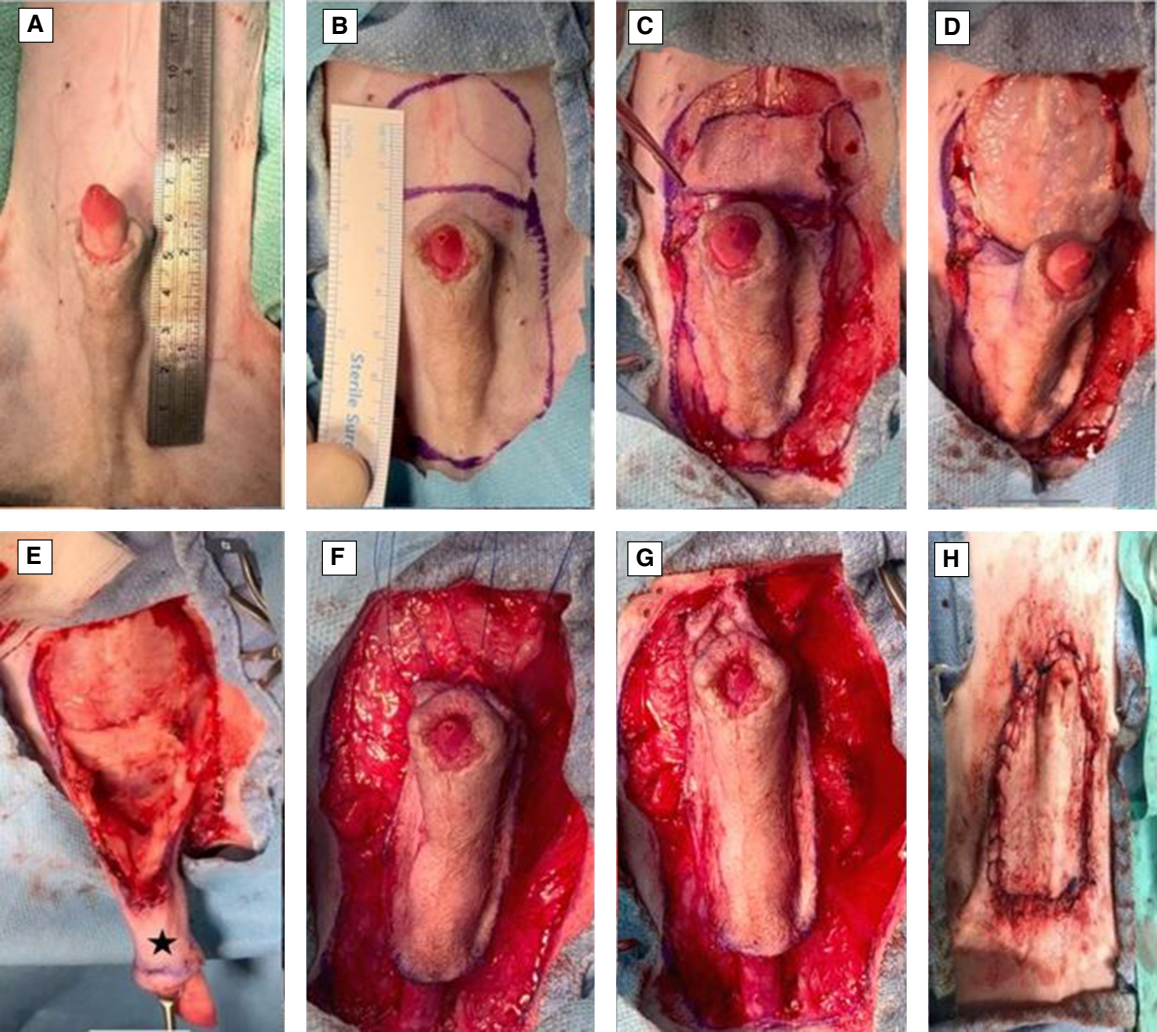
(Dog 2) The ventral abdomen, inguinal and medial thigh regions were clipped and aseptically prepared for surgery on a 2 cm exposed penis due to paraphimosis (A). An inverted U‐shaped incision was marked around the prepuce, with a crescent line above it (B). The skin was incised (C), and subcutaneous tissues were dissected to expose the linea alba and rectus abdominis muscle. The preputial muscles were transected (D). Blunt dissection elevated the prepuce while preserving the caudal superficial epigastric arteries. The prepuce was retracted caudally with forceps (asterisk) (E) and advanced cranially with three horizontal mattress sutures (F), tied sequentially with minimal tension (G). The skin was closed with a continuous subdermal pattern and Ford interlocking sutures (H), achieving complete coverage of the penile exposure

A preputial elevation by skin transection cranial and lateral to the prepuce and cranial translation of the prepuce by transection of the skin caudal to the bulbus glandis (modification 1) as described by Yiapanis et al. (2021) was performed. Under general isoflurane anaesthesia, the dogs were placed in dorsal recumbency, and the ventral abdomen, inguinal and medial thigh regions were clipped and prepared for aseptic surgery. The preputial cavity was flushed with chlorhexidine solution. Using a surgical marker, the proposed incision was drawn as an inverted U‐shaped line surrounding the prepuce. A crescent line cranial to the inverted U‐shaped line was also drawn. The inverted U‐shaped skin incision was made cranial to the prepuce and continued caudally as a para‐median/para‐preputial incision. The para‐median/para‐preputial incisions were joined caudal to the bulbus glandis in the ventral midline, just cranial to the scrotum. The crescent‐shaped skin incision was made cranial to the horizontal part of the inverted U‐shaped incision. The subcutaneous tissues were dissected cranial to the prepuce until the linea alba and rectus abdominis muscle were exposed. The cranial preputial muscles were identified and transected. The skin lateral to the prepuce was sharply incised along the previously drawn lines from the cranial incision to the bulbus glandis caudally. Blunt dissection between the subcutaneous tissues and the external leaf of the rectus abdominis sheath was performed to elevate the prepuce, and care was taken to identify and preserve the blood supply to the peri‐preputial skin (caudal superficial epigastric arteries). In the caudal incision cranial to the scrotum, the penis was freed from its attachments by blunt dissection. Haemorrhage was controlled with diathermy (Led Surtron 200, Led Spa). The prepuce was translated and secured cranially using three 2/0 polypropylene preplaced horizontal mattress sutures inserted between the rectus sheath and the preputial subdermal tissue to achieve full coverage of the penile protrusion. The length of the planned translation was twice the length of the penile protrusion. The middle suture was placed at the level of the ventral midline, and the other sutures were placed laterally to the midline. Cranial preputial translation was achieved by tying the sutures sequentially with minimal tension. Subcutaneous tissues were closed with 4/0 Poliglecaprone sutures in a continuous pattern placed subdermally, and skin was closed in a Ford interlocking pattern using 3/0 Polyamide sutures (Fig 1).

The dogs were hospitalized for 2 days after surgery. During the first 48 hours post‐surgery, the dogs received meloxicam (Metacam, Boehringer Ingelheim) at a dose of 0.1 mg/kg SID IV, tramadol (Tramal, Grunenthal) at a dose of 2 to 5 mg/kg TID IM or buprenorphine (Bupredine, Dechra) at a dose of 10 to 15 μg/kg TID IM. A short‐form Glasgow composite measure pain scale guided the administration of analgesics. Meloxicam was continued orally for another 3 days following surgery. The dogs received cryotherapy (ice packs) on the incision site TID for 24 hours after surgery. An Elizabethan collar was placed and maintained until suture removal 14 days after surgery. Follow‐up was performed by re‐examination at 5 and 14 days after surgery and telephone communication with the referring veterinarian or owner. Minor complications were defined as those that did not require further anaesthesia and surgery for their management. Major complications were defined as those requiring anaesthesia and surgical intervention. The outcome was considered good if a minor complication was noted or excellent if the dog was free of clinical signs.

## RESULTS

### Patient inclusion

Clinical data of the six dogs, including signalment, clinical examination findings, duration of the paraphimosis, length of the penile protrusion, length of the preputial translation at surgery, complications and long‐term follow‐up/outcome, are presented in Table 1.

**Table 1:** Clinical data of the six dogs with idiopathic paraphimosis, which were treated with preputial elevation and translation

Case number	Age (years)	Sex	Breed	Weight (kg)	Clinical examination findings upon presentation	Duration of signs (months)	Length of penile protrusion (cm)	Length of preputial translation (cm)	Short‐term complications	Long‐term complications	Follow‐up (months)	Outcome
1	0.5	MI	German Shepherd	5.0	Permanent penile protrusion	1	2.0	4.0	Incisional serosanguineous discharge	None	36	Excellent
2	4.5	MI	Mixed breed	21.3	Permanent penile protrusion, hyperaemia	24	2.0	4.0	None	None	30	Excellent
3	4.0	MC	Maltese	7.4	Permanent penile protrusion, hyperaemia	1	1.8	3.6	None	None	30	Excellent
4	0.5	MI	Mixed breed	3.7	Permanent penile protrusion, hyperaemia	1	3.0	6.0	Dehiscence, oedema, penile protrusion	None	18	Excellent
5	4.5	MC	Yorkshire terrier	1.9	Permanent penile protrusion, hyperaemia	36	2.5	5.0	Oedema, penile protrusion	Minor penile protrusion	14	Good
6	2.0	MCrypt	Maltese	1.7	Penile protrusion	18	1.0	2.0	None	None	12	Excellent

MC, Male Castrated; Mcrypt, Male Cryptorchid; MI, Male Intact

### Signalment

Breeds included Maltese (*n* = 2), mixed breed (*n* = 2) and one each of German shepherd and Yorkshire terrier. Median age at presentation was three years (range: 0.5 to 4.5 years), and median weight was 4.35 kg (range: 1.7 to 21.3 kg). Three dogs were entire, two were neutered, and one had unilateral left‐sided extrainguinal cryptorchidism.

### Clinical management

Upon presentation, all dogs were clinically healthy with no history of recent mating. Complete blood counts and serum biochemistry analyses were within normal limits. The diagnosis of idiopathic paraphimosis was based on clinical signs and the absence of further penile and preputial abnormalities as documented by plain radiographs of the penis and prepuce and penile/preputial and abdominal ultrasound. In all dogs, the prepuce could be easily moved cranially to cover the protruded penis. The median duration of paraphimosis was 9.5 months (range: 1 to 36 months). The median length of the penile protrusion was 2 cm (range: 1 to 3 cm). The median translation of the prepuce at surgery was 4 cm (range: 2 to 6 cm). In dog 3, the referring veterinarian had partially closed the preputial orifice using a purse‐string suture for 6 days, leading to the resolution of the paraphimosis. However, recurrence occurred 2 weeks after suture removal. In dog 5, the referring veterinarian had made a triangular full‐thickness incision in the mucocutaneous junction at the ventral prepuce in an attempt to widen the preputial orifice without success. In dog 5, the triangular defect at the ventral prepuce was closed with interrupted sutures in two layers: 4/0 poliglecaprone for the subcutaneous tissues and 3/0 polyamide for the skin. In dog 6, removal of the extrainguinal cryptorchid testicle was performed through the lateral preputial incision. Orchidectomy of the left descended testicle was performed 2 months after surgery.

### Postoperative complications

**FIG 2:**
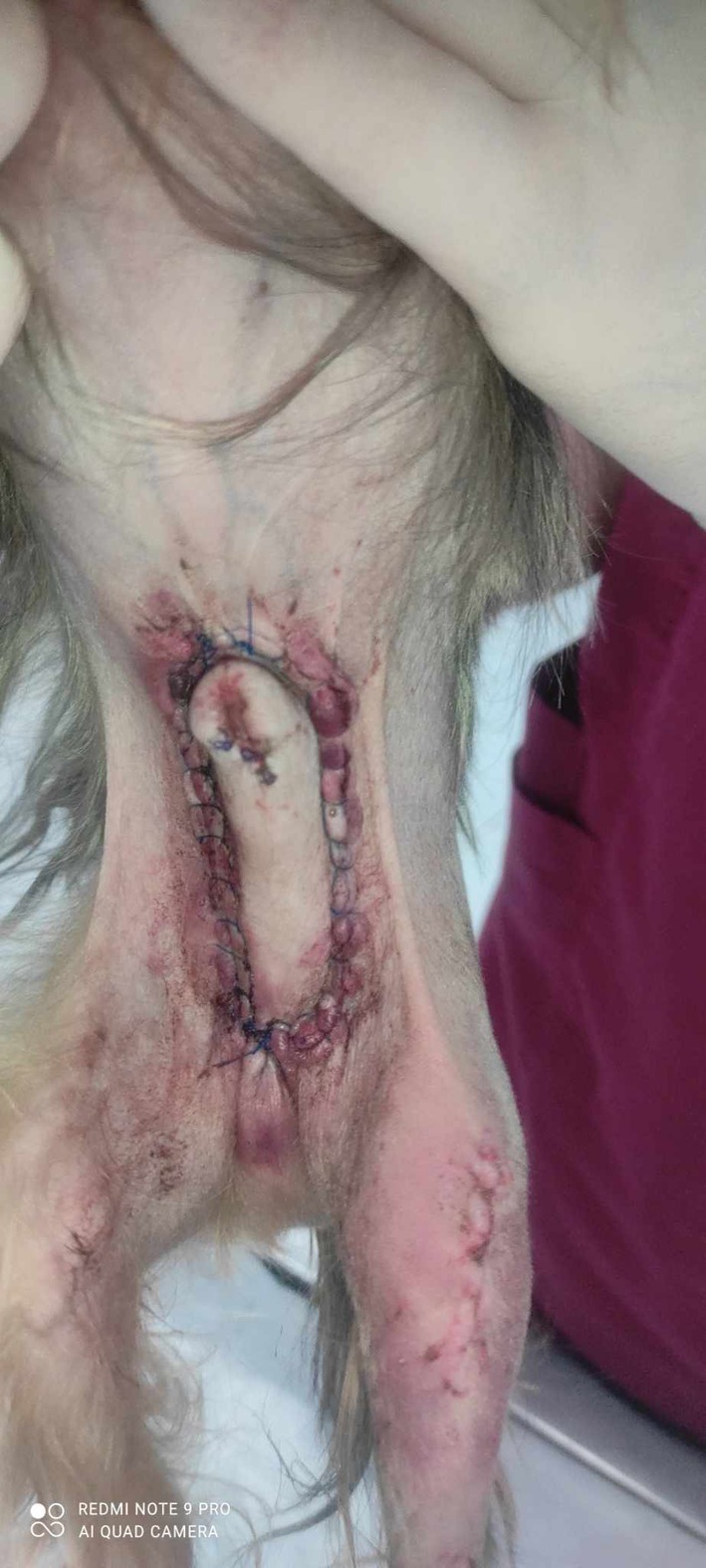
(Dog 5) Preputial oedema and redness surrounding the incision developed within 48 hours after surgery

**FIG 3:**
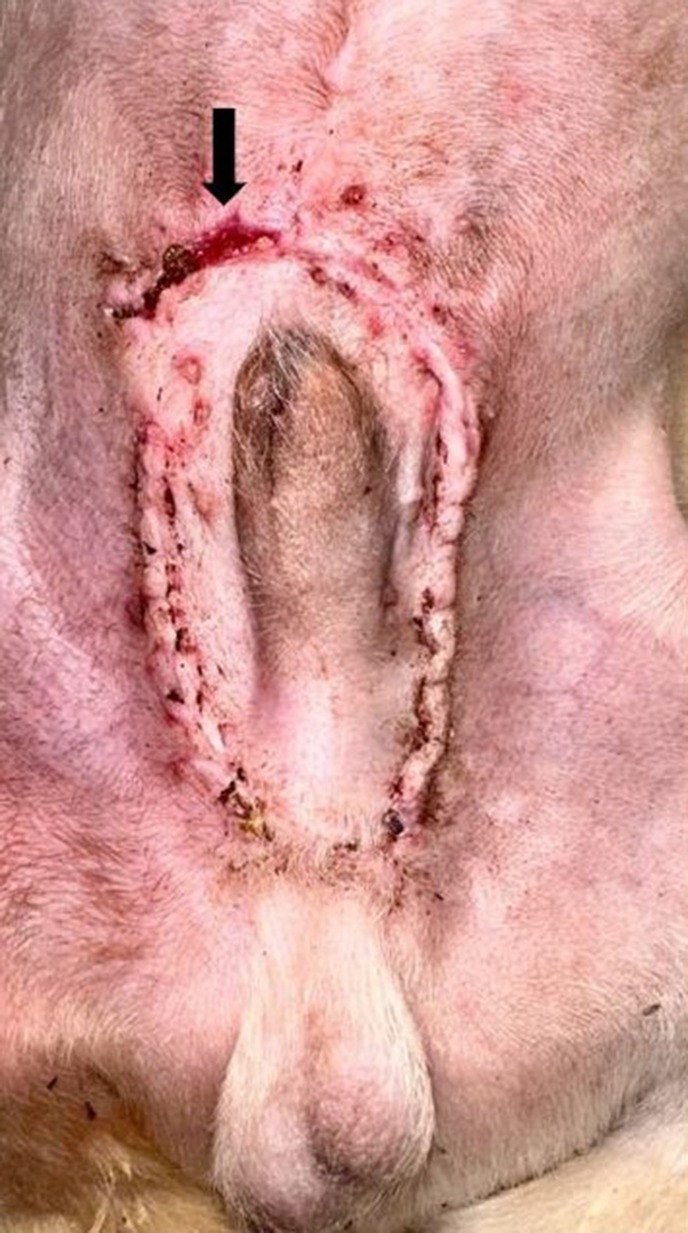
(Dog 4) A 1.5 cm dehiscence was observed on the cranial right side of the inverted U‐shaped incision (arrow). The dehiscence was left to heal by second intention

**FIG 4:**
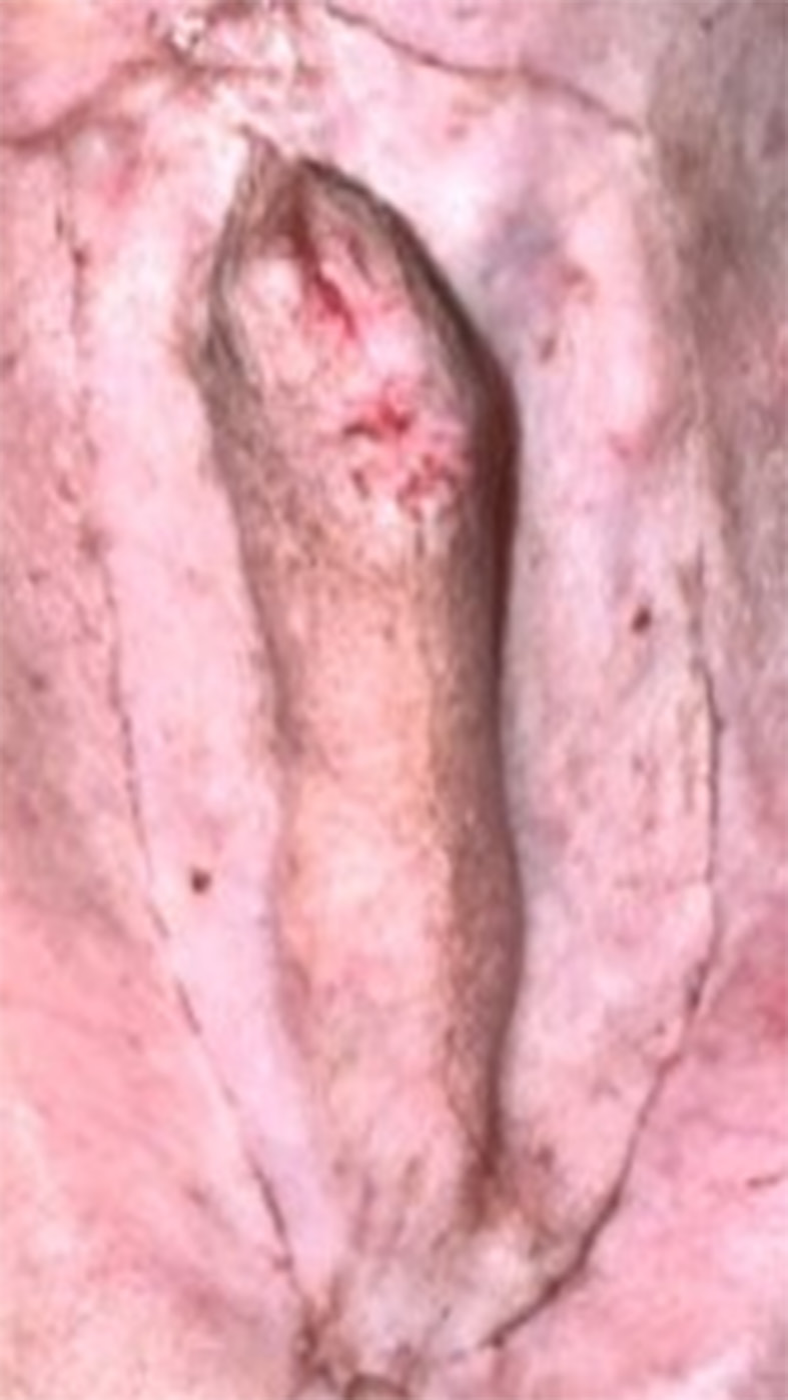
(Dog 5) The surgical wound was completely healed 1 month after surgery and oedema was resolved. No paraphimosis was observed

All dogs had a smooth recovery from anaesthesia. No postoperative complications were observed in 3/6 dogs. In dog 1, a small amount of serosanguineous discharge was observed to exit from the cranial incision, which resolved within 24 hours without intervention. In dogs 4 and 5, oedema formation was seen in the prepuce, which resolved with ice packs TID for the first 24 hours, followed by hot packs TID for another 2 days (Fig 2). In these two dogs, mild penile protrusion of less than 0.5 cm was reported, accompanying the oedema, which also resolved as soon as the oedema subsided. In dog 4, a 1.5 cm dehiscence on the right side of the inverted U incision was reported, which was allowed to heal by second intention (Fig 3). All dogs were re‐examined 5 and 14 days after surgery at suture removal and were clinically healthy with no signs of paraphimosis (Fig 4). Median follow‐up time was 24 months (range: 12 to 36 months). Five out of six dogs had an excellent outcome. In dog 5, at re‐examination 14 months after surgery, a 0.5 cm long intermittent penile protrusion was reported, but it was considered mild with no clinical significance, and no revision surgery was deemed necessary.

## DISCUSSION

In the study presented here, a modified preputial elevation and cranial translation of the prepuce by transection of the skin caudal to the bulbus glandis was successfully used for the treatment of idiopathic paraphimosis in six dogs. Complications following surgery were infrequent and did not require surgical revision.

Cranial advancement of the prepuce has been shown to successfully cover the exposed penis in four dogs with idiopathic paraphimosis with an exposure <1.5 cm (Papazoglou, 2001). In this study, recurrence was detected in two dogs that had an increased length of penile exposure (2.2 and 2.4 cm, respectively). Recurrence was noted in dogs: one with a 2.5 cm (long‐term) and one with a 3 cm (transient) penile protrusion. However, we feel that outcomes in our study were superior to a prior study (Papazoglou, 2001). Division and reapposition of the paired preputial muscles, along with the inclusion of the external leaf of the rectus sheath, during suturing the cranial dermal border of the prepuce, have been advocated to reduce the tension across the incision line and decrease the possibility of dehiscence (Papazoglou, 2001). Failure of the preputial muscles to keep the prepuce in its advanced position was the likely cause of the recurrences seen in this study. The use of polyglactin 901 suture material with a loss of tensile strength within 2 weeks may have contributed to caudal relaxation of the advancement over time, leading to a recurrence.

Recently, a cadaveric study in 10 dogs addressed longer exposures of the penile paraphimosis. In this study, cranial translation of the elevated prepuce from the ventral abdominal wall before and after (1) releasing the skin caudal to the prepuce (modification 1) and (2) the attachment of the penile lamina interna (modification 2) using three preplaced sutures between the prepuce and the linea alba has been reported (Yiapanis et al., 2021). In this study, the median maximum cranial preputial translation after preputial elevation and after modifications 1 and 2 was 15 mm, 29.5 mm and 39 mm, respectively.

In our study, the paired preputial muscles were transected to allow preputial elevation from the abdominal wall, but no suturing was performed. Following preputial elevation from the ventral abdominal wall, a skin incision around the prepuce and cranial to the scrotum allowed for the release of preputial skin and its cranial translation. The placement of three horizontal mattress sutures of polypropylene was undertaken to anchor the prepuce on the external sheath of the rectus abdominis, decreasing tension and providing long‐term support to the translated prepuce. Our technique differs from the technique reported by Yiapanis et al. (2021), where one simple interrupted suture was used between the prepuce and linea alba to achieve adequate preputial translation. The median translation length of our technique after tightening the sutures was greater than the median translation of the Yiapanis et al. (2021) technique after modification 1. By using our technique, complete coverage was achieved even in dog 4 with a 3 cm penile exposure. Damage to the caudal superficial epigastric vessels during transection of the skin caudal to the bulbus glandis could result in significant skin necrosis, dehiscence and wound complications. Closure of the caudal defect was achieved with placement of a tension‐relieving continuous suture placed subdermally. Tension on the caudal aspect of the preputial skin defect may also be managed using an H‐plasty or a 90° transposition flap as described by Schreiber et al. (2023). Skin closure in our study was achieved using the Ford Interlocking pattern, as this type of suture provides quicker closure, better skin apposition and even tension distribution along the wound edges compared to simple continuous and simple interrupted sutures. In the present study, dogs 1, 2, 4 and 6 were intact males, but no orchiectomy was performed at the time of surgery. This was because a preputial incision cranial to the scrotum for orchiectomy could interfere with the caudal preputial incision and advancement; besides, no benefit from orchiectomy to the management of idiopathic paraphimosis has been reported (Papazoglou, 2001).

Postoperative complications from preputial elevation and cranial translation were minimal for all dogs in our study. Two dogs developed oedema that was attributed to surgical trauma to the lymphatic vessels. The development of oedema was accompanied by slight penile protrusion of less than 0.5 cm, which resolved after conservative treatment. Transient penile exposure following preputial oedema and swelling in two dogs was suspected to occur due to a small space within the preputial cavity to the extent that it could not accommodate penile retraction within the prepuce (Sprayberry & Lu, 2021). Further, the postoperative resolution of oedema led to a reduction in penile protrusion. Long‐term follow‐up of the dogs revealed recurrence of paraphimosis in dog 5. Penile exposure in this dog was minimal, and no further treatment was necessary. The cause of penile exposure cannot be easily determined. In this dog, as there was longer exposure (2.5 cm) we might have considered performing a more cranial translation of the prepuce following dissection of the penile mucosa away from the bulbus glandis (modification 2), without entering the preputial cavity; this might have contributed to the complete coverage of the exposed penis as reported by Yiapanis et al. (2021).

Cranial translation of the elevated prepuce can be successfully used for the treatment of long‐length penile exposure (>1.5 cm) in idiopathic paraphimosis in dogs. Complications following surgery were infrequent and did not require surgical revision.

## Author contributions

**I. P. Thyriakis:** Conceptualization (equal); data curation (lead); methodology (equal); writing – original draft (lead). **C. Ververidis:** Conceptualization (equal); methodology (supporting); writing – review and editing (equal). **L. Pavlidis:** Conceptualization (equal); methodology (supporting); writing – review and editing (equal). **V. Angelou:** Conceptualization (equal); data curation (supporting); methodology (supporting). **K. Chatzimisios:** Conceptualization (equal); data curation (supporting); methodology (supporting). **L. G. Papazoglou:** Conceptualization (lead); methodology (lead); data curation (supporting); writing – review and editing (lead).

## Conflict of interest

The authors declare no financial or personal conflict of interest.

## Data Availability

The data that support the findings of this study are available on request from the corresponding author. The data are not publicly available due to privacy or ethical restrictions.
